# Editorial: New perspectives on the role of vision in sports

**DOI:** 10.3389/fspor.2025.1582761

**Published:** 2025-03-11

**Authors:** Clara Martinez-Perez, Cristina Alvarez-Peregrina, Miguel Ángel Sánchez-Tena

**Affiliations:** ^1^ Aeronautical, Tourism and Aviation Department, School of Business, Engineering and Aeronautics, ISEC Lisboa, Instituto Superior de Educação e Ciências, Lisbon, Portugal; ^2^ Optometry and Vision Department, Faculty of Optics and Optometry, Complutense University of Madrid, Madrid, Spain

**Keywords:** oculomotor, sports vision training, visual perception, visuomotor coordination, athletic performance

**Editorial on the Research Topic**
New perspectives on the role of vision in sports

Sports vision training has emerged as a crucial component in the optimization of athletic performance. Bibliometric data indicates a significant increase in research on this topic over the past decade. In 2015, there were approximately 118 publications, whereas by 2023, this number had more than doubled to 260, peaking at 327 in 2021. The ability to process visual information efficiently and integrate it with motor responses is fundamental in many sports, where quick reactions and precise coordination are required. Vision plays a key role in decision-making, anticipation, and reaction time, elements that are essential in different sports. Recent advancements have demonstrated that targeted vision training interventions can significantly enhance an athlete's perceptual and cognitive abilities, leading to improved performance across various disciplines (1).

Sports vision training has evolved into a key area of research, with various studies exploring its impact on athletic performance. A citation network analysis conducted by Nascimento et al. (1), provided an in-depth overview of the main research areas in this field, identifying four major clusters: ocular injuries, visual training methods and efficiency, visual fixation training, and concussions. This study highlighted the growing interest in understanding how visual skills contribute to sports performance and the effectiveness of different training approaches in enhancing athletes' perceptual and cognitive abilities.

Given the increasing recognition of the role of visual skills in athletic success, researchers have sought to develop and refine specific training techniques tailored to different sports disciplines. Building on this framework, recent studies have focused on specific training techniques to optimize visual function in different sports. Vasile and Stănescu, demonstrated that strobe training can enhance proprioceptive awareness and spatial cognition in climbers by limiting visual input. Wu et al., examined the role of depth perception, reaction time, and eye-hand coordination in boxing, showing that superior visual processing speed correlates with improved punch accuracy. Similarly, Guo et al., analyzed a structured vision training program designed to improve skeet shooting accuracy, emphasizing the importance of visuomotor coordination. Additionally, Limballe et al., investigated how virtual reality (VR) can be used to manipulate visual conditions in boxing training, revealing that athletes develop adaptive gaze strategies to maintain performance under altered conditions. These studies provide valuable insights into how vision training techniques can be incorporated into athletic training regimens to enhance performance in competitive sports.

## Enhancing spatial awareness and reaction time

One of the fundamental findings across these studies is the significant role of vision training in improving spatial awareness and reaction time. In climbing, where athletes must navigate complex vertical environments, Vasile and Stănescu demonstrated that strobe training enhances an individual's ability to process movement-related cues with minimal visual input. Their study showed that climbers who underwent 20 sessions of strobe training exhibited significant improvements in cognitive agility, spatial mapping, and upper-body coordination. These findings suggest that vision training can help athletes refine their proprioceptive skills, allowing them to make more precise and calculated movements even when visual conditions are compromised.

A similar pattern was observed in boxing, where rapid decision-making and split-second reactions are crucial for competitive success. Wu et al. found that athletes with superior visual processing speed and depth perception performed better in punch accuracy tests. The study indicated that vision training targeting reaction time and peripheral awareness could improve a boxer's ability to anticipate opponent movements and respond more effectively to dynamic fight scenarios. These results highlight the importance of integrating vision exercises, such as rapid gaze-shifting drills and peripheral vision expansion techniques, into combat sports training to enhance overall performance.

## Optimizing visuomotor coordination in precision sports

In sports that require high levels of visuomotor coordination, such as skeet shooting, targeted vision training can significantly enhance performance. Guo et al. conducted a six-week intervention that focused on improving visual tracking, near-far quickness, and hand-eye coordination. The results revealed that athletes who participated in vision training achieved higher shooting accuracy and faster target acquisition times compared to those in the control group. These findings reinforce the role of sports vision training in refining sensorimotor integration, allowing athletes to execute precise motor actions based on visual cues.

The integration of virtual reality (VR) in sports training provides new avenues for enhancing visual adaptability. Limballe et al. investigated the effects of gaze-contingent blur in a VR boxing simulation, where visual conditions were artificially altered to challenge an athlete's ability to track opponents and execute precise strikes. Interestingly, their findings showed that despite the imposed visual limitations, boxers adapted their gaze behaviors and maintained performance levels, suggesting that exposure to controlled visual perturbations can improve an athlete's resilience to unpredictable visual environments. This study underscores the potential of VR as a training tool for developing advanced gaze strategies and perceptual flexibility in competitive athletes. However, some limitations remain, including the need for standardized protocols, individual variability in adaptation, and unknown long-term effects. In boxing, further studies should assess VR-based gaze training in real fight conditions; in climbing, strobe training may require age-specific adaptations whereas in shooting sports, refining visuomotor programs could enhance precision and reaction times.

## Conclusion

The findings from these studies highlight the importance of vision training in enhancing sports performance across various disciplines. Improvements in spatial awareness, reaction time, and visuomotor coordination contribute significantly to athletic success. Strobe training, structured vision exercises, and VR-based adaptations demonstrate the versatility of vision training in optimizing performance. To maximize these benefits, coaches and athletes should incorporate vision training into regular practice routines, tailoring exercises to sport-specific demands. Future research should further explore the long-term benefits of these interventions and their practical integration into standard training protocols. By advancing sports vision research, athletes can refine their perceptual skills and gain a competitive edge in high-performance sports.
